# CNT–TiO_2_ core–shell structure: synthesis and photoelectrochemical characterization†

**DOI:** 10.1039/d1ra05723e

**Published:** 2021-10-08

**Authors:** Vasu Prasad Prasadam, Ali Margot Huerta Flores, Naoufal Bahlawane

**Affiliations:** Material Research and Technology Department, Luxembourg Institute of Science and Technology Rue du Brill L-4422 Belvaux Luxembourg naoufal.bahlawane@list.lu

## Abstract

Porous composite coatings, made of a carbon nanotube (CNT)–TiO_2_ core–shell structure, were synthesized by the hybrid CVD-ALD process. The resulting TiO_2_ shell features an anatase crystalline structure that covers uniformly the surface of the CNTs. These composite coatings were investigated as photoanodes for the photo-electrochemical (PEC) water splitting reaction. The CNT–TiO_2_ core–shell configuration outperforms the bare TiO_2_ films obtained using the same process regardless of the deposited anatase thickness. The improvement factor, exceeding 400% in photocurrent featuring a core–shell structure, was attributed to the enhancement of the interface area with the electrolyte and the electrons fast withdrawal. The estimation of the photo-electrochemically effective surface area reveals that the strong absorption properties of CNT severely limit the light penetration depth in the CNT–TiO_2_ system.

## Abstract 2

CNT–TiO_2_ core–shell nanostructured coatings were made using a hybrid CVD/ALD process. The evaluation of these films as photoanodes for the photoelectrochemical water splitting reaction reveals a clear benefit from the involvement of CNTs.

## Graphical abstract

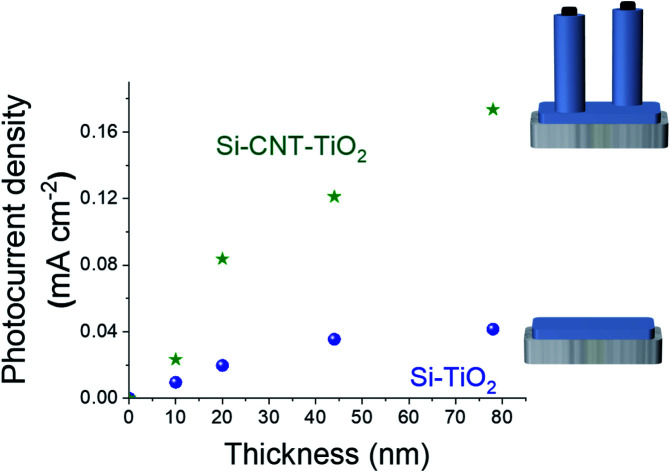

## Introduction

Photo-electrochemical (PEC) water splitting is an appealing approach for clean hydrogen energy generation.^1^ Hereby, the process is essentially limited by the water oxidation reaction, which drives intense research for the development of high-performance anode materials. In this context, non-oxide semiconductors feature convincing performances,^2^ however, they are chemically unstable in acidic and alkaline environments.^3,4^ In contrast, several metal oxides exhibit a better chemical stability in the dark and under illumination.^5^ Furthermore, metal oxide semiconductors come with additional merits such as the cost-effectiveness, non-toxicity and high abundance. In this context, TiO_2_, ZnO, WO_3_, Fe_2_O_3_ and BiVO_4_ have been intensively investigated in PEC water splitting.^6,7^

Among the available metal oxides, TiO_2_ has a high abundance and high chemical stability.^8^ Titanium oxide features eight polymorphs, among which anatase and rutile have shown a significant photocatalytic activity towards water splitting.^9,10^ Anatase–rutile composite forms a heterostructure where charge carrier separation is improved, and the bandgap is decreased. As a result, the composite significantly outperforms the photocatalytic property of the individual constituents.^11^ TiO_2_ has a suitable positioning of the conduction and valence band energies to drive hydrogen evolution (HER) and oxygen evolution reactions (OER). This is associated with a band gap in the UV (3.0–3.2 eV), which limits the theoretical efficiency. Furthermore, the low charge carrier mobility with short diffusion length (10–100 nm)^12^ imposes either a reduction of its thickness to match the diffusion length scale, or nano-structuring.^13^ Further improvements were reported using several approaches such as, doping, forming a heterojunction with other semiconductors and by the application of a co-catalyst.^14–16^ Nano-structured TiO_2_ is synthesised by different processes such as hydrothermal, solvothermal, titanium-foil anodization and template-assisted process.^14,15^

The poor electrical conductivity of TiO_2_ nano-structures and the fast recombination of photogenerated charges limit the PEC water splitting performance,^17^ and the addition of CNT has a beneficial effect. The electron transfer is energetically favourable from the TiO_2_ conduction band to the CNT π-system.^18^ So far, CNT–TiO_2_ structures are synthesised by sol–gel^19,20^ and hydrothermal processes,^21,22^ which are affected by the challenging CNT dispersion in aqueous media as the unmodified CNTs are hydrophobic. Therefore, the process is difficult to control and heat treatments are needed to enhance the crystallization of TiO_2_.^23^ The electronic structure of the CNT–oxide interface is degraded due to the chemical modification of the CNT surface, a step that is necessary to enable their appropriate dispersion.^20,24^ The presence of TiO_2_ as nanoparticles decorating the CNT surface leads to charges recombination upon interaction with the electrolyte.^25^ The performance of TiO_2_–CNT coatings made by hydrolysis results in a photocurrent density of 0.05 mA cm^−2^ at 1.6 V_RHE_,^21^ while the core–shell CNT–TiO_2_ synthesised by gas phase process has shown a photocurrent density of 0.16 mA cm^−2^ at 1 V_RHE_.^26^ In the latter case the CNTs were grown at 750 °C, detached and drawn on the PEC surface prior to the deposition of TiO_2_.^26^ Here we propose a simple and one-pot gas-phase process, low temperature hybrid CVD-ALD, for the synthesis of an innovative CNT–TiO_2_ core–shell structure, for which the photo-electrochemical properties are investigated.

## Materials and methods

The synthesis of the CNT–TiO_2_ core–shell film architecture involves a single-pot hybrid Chemical Vapor Deposition-Atomic Layer Deposition (CVD-ALD) process. The deposition of carbon nanotube on silicon substrates was performed using thermal CVD. An equimolar ethanol solution, 0.65 × 10^−3^ mol L^−1^, of cobalt acetylacetonate (Co(acac)_2_) and magnesium acetylacetonate (Mg(acac)_2_) was implemented as a single precursor feedstock. This feedstock was introduced into the reactor *via* an evaporation cylinder at 220 °C, using a pulsed spray with a frequency of 4 Hz and using the opening time of 4 ms. The deposition was run for 2 h at 10 mbar, using a substrate temperature of 485 °C. The thickness of the film was assessed *via* the cross-section SEM inspection, and the density was assessed gravimetrically.

The ALD of the TiO_2_ shell around the individual CNTs was achieved using the alternated surface exposure to titanium tetra-isopropoxide (TTIP) and water vapor. Here an ALD cycle consists of 4 steps: TTIP/purge/H_2_O/purge, and the growth rate is defined by the deposited thickness of TiO_2_ per ALD cycle (growth per cycle/GPC). Both precursors were maintained at room temperature during the process, which conveniently limits their eventual condensation in the transport lines. The deposition pressure was adjusted at 0.5 mbar, while the temperature and the exposure times were a subject of a systematic study. The thickness of TiO_2_ films on planar silicon was measured using a multi-wavelength Ellipsometer (Film Sense) with the Cauchy Model.

X-ray diffraction (Bruker D8), with Cu-K_α_ as the X-ray source, was used to identify the present crystalline phases. Here, the data were collected in the grazing incidence mode 0.5° while scanning the detector from 0° to 90° with a step size of 0.02°. Raman scattering was performed using an InVia Raman spectrometer from Renishaw with a 633 nm laser and a power density of 87 μW cm^−1^.^2^

The CNT–TiO_2_ structure was characterized using transmission electron microscopy (S/TEM Themis Z G3, 300 kV, Thermo Fisher Scientific). The elemental mapping was performed using a combined EDX (energy dispersive X-ray spectrometer) analysis and high-angle annular dark-field scanning transmission electron microscopy (HAADF-STEM, 29.5 mrad, probe corrected). The coated CNTs were sampled, by scratching the surface, and deposited on lacey carbon grids. The morphology of the films was inspected using the FEI Helios Nanolab 650 scanning electron microscope (SEM) at a working distance of 5 mm and using an acceleration voltage of 5–10 kV.

A standard three-electrode setup was used for the photoelectrochemical measurements with the Si–CNT–TiO_2_ or Si–TiO_2_ as the working electrode. All voltages were measured *versus* Ag/AgCl reference electrode, and platinum (Pt) was used as the counter electrode. The electrolyte was an aqueous solution of 0.1 M NaOH (pH = 12.7). All the potentials from Ag/AgCl reference were converted to RHE based reference throughout eqn (1).

1 *E*_RHE_ = *E*_Ag/AgCl_ + 0.059 × pH + 0.1976

The electrode area, 2 cm^2^, was front-illuminated using a Xe-lamp at 100 mW cm^−2^. Electrochemical measurements were conducted using an AUTOLAB potentiostat. Steady state current–voltage curves were used for assessing the electrochemical performance, whereas the AC impedance spectroscopy provided information on the contribution of various resistive losses (polarization and ohmic/ionic) to the performance of the photoanodes.

## Results and discussion

### CVD of CNT films

**Fig. 1:**
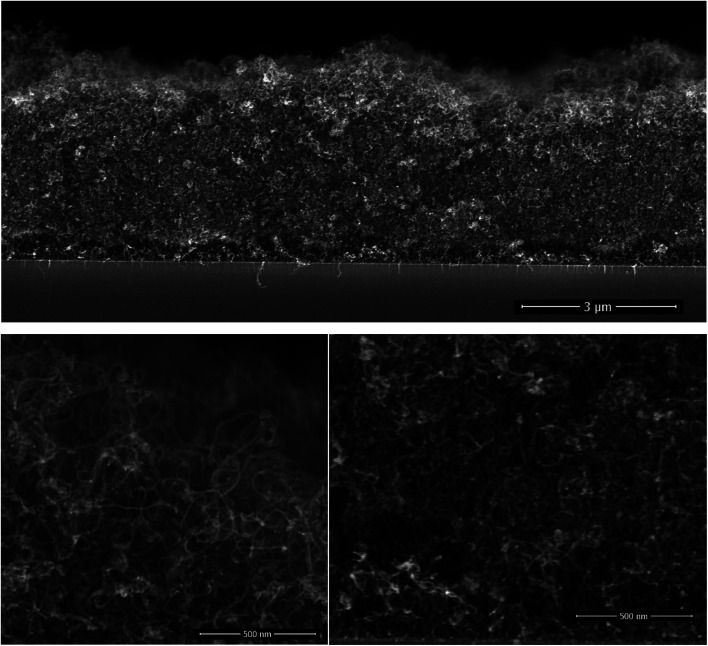
The cross-section morphology of a ∼4 μm thick film of randomly oriented CNT. The high magnification micrographs at the surface (bottom left) and at the interface (bottom right) illustrate the homogeneous entangled CNTs, featuring an outer diameter of 12 nm.

The CNT growth is performed in a single step using a single feedstock approach. In this process ethanol vapor is thermally converted to CNTs *via* the mediation of *in situ* formed catalyst and promoter. The *in situ* reaction of ethanol with transition metal acetylacetonates at moderate temperatures yields metallic nanoparticles,^27–29^ that catalyze the CNT growth; whereas, the thermal decomposition of magnesium acetylacetonate yields MgO, which the basicity promotes the CNT growth at temperatures exceeding 330 °C.^30^ The resulting films are composed of randomly oriented multi-wall CNTs featuring an average outer diameter of (12 ± 0.6) nm as assessed by SEM inspection.^30^ The inner/outer diameter of the CNTs were confirmed by TEM at 5 nm/12 nm along with the existence of 8 graphene layers.^30^ The grown 4 μm thick CNT film on interdigital electrodes features an electrical resistance of 5 Ω. Such a low electrical resistance results from the strong crosslinking between the MWCNTs. The cross-section morphology of the grown film on silicon substrates, Fig. 1, displays a porous CNT structure for which the density is gravimetrically estimated at 0.4–0.6 mg cm^−3^. This density is at least three orders of magnitude lower relative to densely packed CNTs.^31^ Although the geometric thickness of the film is homogeneous throughout the substrate, the CNTs occupy a marginal volume fraction.

A close inspection at the surface of the CNT film and at the interface with the silicon substrate shows a similar morphology, which is a consequence of the simultaneous introduction of the catalyst and promoter along the deposition process. Cobalt and magnesium were found to be distributed homogeneously across the thickness of the film and their content in the CNT film was estimated using EDX at 4 at% = Co/(C + Co + Mg), and 9 at% = Mg/(C + Co + Mg). It is worth mentioning that the presence of cobalt might contribute to the electrochemical behaviour of non-coated CNTS.

The as-grown CNT films fail in the adhesion scotch test, as the CNTs are easily detached from the substrate, and they partially detach from the surface when dipped in the electrolyte under sonication. This limitation was overcome *via* the conformal deposition of metal oxides around CNTs to form a core/shell structure.^32^ In this context, shells of aluminum oxide or silicon oxide were investigated. Here we do investigate the ALD of TiO_2_ around the CNTs to provide them the ability to split water in a photoelectrochemical setup. It is worth mentioning that the CVD of CNTs and their coverage with an oxide shell layer can be performed in the same reactor, and the resulting films are mechanically robust and remain unaffected when ultrasonicated or dipped in the electrolytes.

### ALD of TiO_2_

**Fig. 2:**
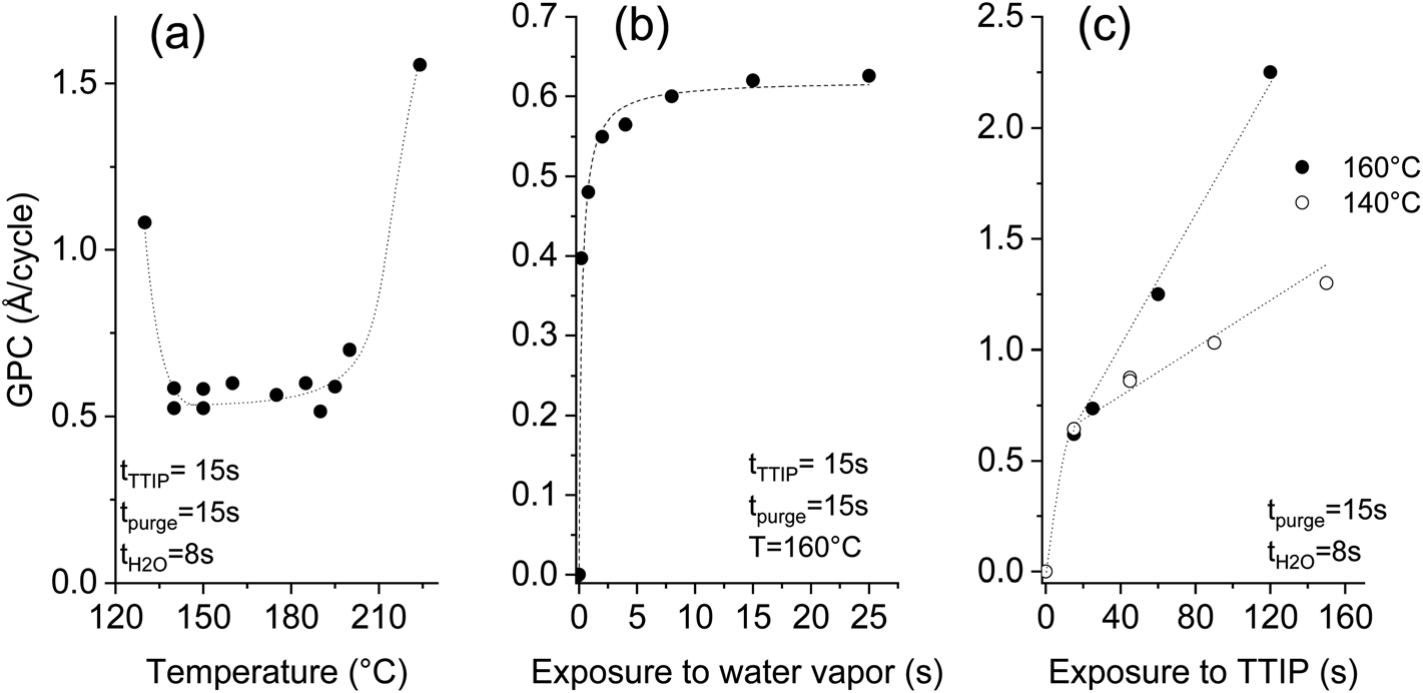
Effect of the temperature (a), water (b) and TTIP (c) exposure times on the deposited thickness per cycle at a pressure of 0.5 mbar (dashed lines are used to guide the eyes).

A systematic study was performed on silicon substrates to establish conditions where the thermal ALD of TiO_2_ can be performed. For the investigation of the temperature effect, Fig. 2a, the deposition recipe involved a surface exposure time of 15 s to TTIP, and 8 s exposure to water vapor, both are separated by 15 s of purge using 50 sccm of argon. The impact of the surface temperature on the growth per cycle (GPC) is marginal in the 140–195 °C temperature range. A rise of the GPC, outside this range, is associated with the dominant thermal decomposition at high temperature and the plausible insufficient purging of water vapor at low temperature. A GPC of 0.56–0.58 Å per cycle was measured in the plateau, which agrees with the ∼0.5 Å per cycle reported for the hydrolysis of TTIP at 250 °C.^33^ The same ALD chemistry was implemented at 80–120 °C (ref. 34) and 160 °C,^35^ and GPCs of 0.33 and 0.68 Å per cycle were reported respectively. The diverging literature data regarding the GPC values might hint at the presence of competing deposition mechanisms.

Beyond the great relevance of self-limited reactions for the attainment of conformal coatings on structures with high aspect ratios, such as CNTs, studying the effect of the exposure time helps to understand the ALD process. Investigating the effect of the surface exposure time to water vapor was performed at a deposition temperature of 160 °C, while maintaining the TTIP exposure time at 15 s. The displayed results in Fig. 2b evidence the self-limited hydrolysis reaction step. An exposure time of 8 s is appropriate to completely hydrolyze the adsorbed TTIP, which enables a maximal GPC of ∼0.6 Å per cycle. A non-complete hydrolysis at short exposures to water vapor leaves ligand moieties that poison the surface and yield a reduced GPC.

Unlike the self-limited behavior observed for the hydrolysis reaction, the TTIP adsorption gives a continuous increase at 160 °C as displayed in Fig. 2c. A strong rise of the GPC with the TTIP exposure time is observed, reaching 2.3 Å per cycle after 120 s. No saturation was observed, which indicates a significant CVD contribution. In this case, the thermolysis of TTIP leads to the growth of TiO_2_ film even in the absence of water vapor.

Decreasing the deposition temperature from 160 °C to 140 °C significantly limited the rise of the GPC with the TTIP exposure; but did not suppress it. Decreasing the deposition temperature would logically further limit the contribution of the CVD components, and likely enable the ALD-typical self-limited growth. Nevertheless, it is worth mentioning that 140 °C is at the low temperature side of the processing window featuring a constant GPC (Fig. 2a). Therefore, the CVD growth contribution persists in the optimized pseudo-ALD process. The omnipresent CVD contribution might be the reason behind the diverging literature data regarding the reported values of the GPC. The occurrence of a competing CVD pathway was demonstrated below the TTIP thermolysis temperature.^36–38^ This behavior was attributed to the catalytic effect of the under-coordinated Ti^+4^, which is assumed to induce the dehydration of TTIP or of the formed isopropanol.^36,37^ As a result, further growth occurs instead of a surface saturation upon exposure to TTIP. Dosing isopropanol onto a surface of TiO_2_ (110) shows that the associative dehydration reaction extends from 30 to 180 °C.^39^ Therefore, only a pseudo-ALD of TiO_2_ can be expected from the hydrolysis of TTIP; nevertheless, limiting the surface exposure to TTIP would reduce the CVD contribution.

### CNT–TiO_2_ core–shell structure

**Fig. 3:**
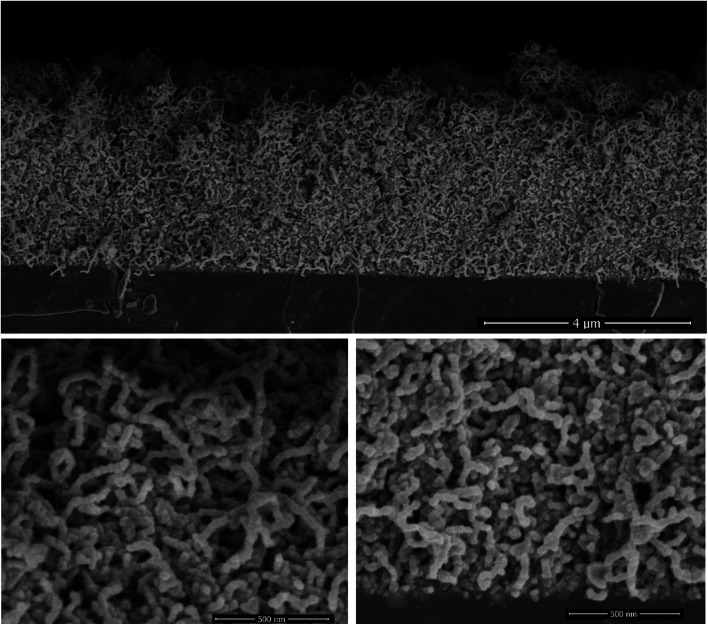
The cross-section morphology of a ∼4 μm thick film of randomly oriented CNT after the deposition of a TiO_2_ shell at 160 °C. The ALD was performed using 8 s and 15 s as exposure times for water and TTIP, while the purge time was fixed at 15 s. The high magnification micrographs at the surface (bottom left) and at the Si–CNT interface (bottom right) illustrate the homogeneous entangled CNT–TiO_2_ core–shell, featuring an outer diameter of 35 nm.

The ALD of TiO_2_ was performed on the CNT layers within the identified temperature window (140–200 °C). The SEM cross-section displayed in Fig. 3 corresponds to a film grown at 160 °C. At first glance, the initial porous structure of the randomly oriented CNT is retained after the deposition of TiO_2_. The apparent diameter of the CNT is however significantly larger, 35 nm, relative to the non-coated CNTs (12 nm), and their surfaces feature faceted crystallites. The outer diameter hints at the deposition of a shell with a thickness of 11.5 nm around the CNT core after 200 cycles. This corresponds to a GPC of 0.58 Å per cycle, which is comparable to the growth on planar silicon (Fig. 2). The resulting morphology was further inspected across the thickness of the CNT layer. It is worth highlighting that a slightly higher CNT density is observed at the interface with silicon for the as-grown films. The surface and interface regions, Fig. 3, reveal an identical morphology, and the coated CNTs feature a similar diameter across the layer, which is a strong evidence about the conformality of the TiO_2_ coating. The further densification of the CNT–TiO_2_ layer at the interface is an additional evidence of the ability of the ALD to enable a full infiltration.

**Fig. 4:**
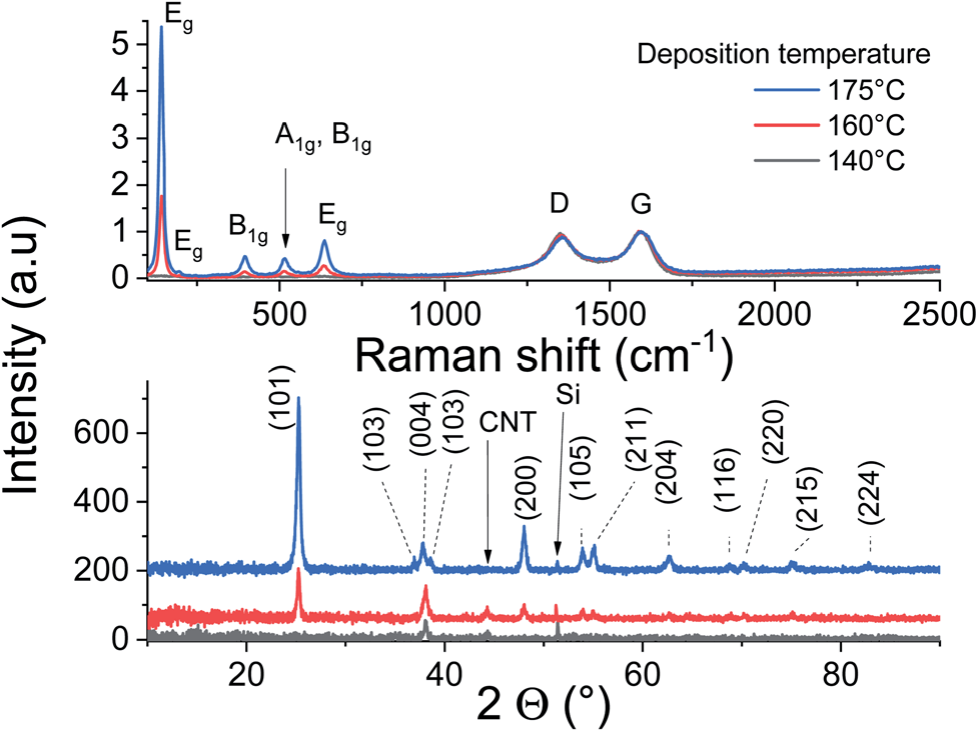
Raman scattering and XRD analysis of CNT–TiO_2_ core–shell.

Raman scattering and X-ray diffraction on the ALD-grown titanium oxide over CNTs at 140, 160 and 175 °C are displayed in Fig. 4. It is worth mentioning that these films have the same thickness, as the GPC in these temperature conditions is similar, but their analyses show a significant contrast. The CNT characteristic Raman bands at 1345 cm^−1^ (D band) and 1589 cm^−1^ (G band) are observed with a low *I*_G_/*I*_D_ ratio for all samples, which was associated with the presence of defects at the outer graphene layer of the MW-CNT.^30^ The anatase fingerprint is only observed for films grown at 160 °C and 175 °C. The most intense and sharp peak at ∼140 cm^−1^ in addition to the peaks at ∼200 and 630 cm^−1^ are attributed to the E_g_ modes.^40^ The peak at 395 cm^−1^ was assigned to a B_1g_ mode, whereas the peak at 513 cm^−1^ involves components from A_1g_ and B_1g_.^40^ Relative to films grown at 175 °C, the signals are weaker for the films grown at 160 °C, while no peaks can be distinguished for the films grown at 140 °C.

The performed XRD analysis confirms the polycrystalline nature of the grown film at 175 °C. The recorded peaks in the XRD pattern correspond to anatase TiO_2_ (pdf 01-075-2547). The film grown at lower temperatures show weak peaks intensities of the same phase, indicating their poor crystallinity. The thermal activation during deposition favors the atoms surface-diffusion towards sites with minimized energy. Therefore, the crystallization process improves to reach saturation at sufficiently high temperatures. Weak diffraction peak corresponding to Si substrate and CNT can be identified.

### Photoelectrochemical measurement

**Fig. 5:**
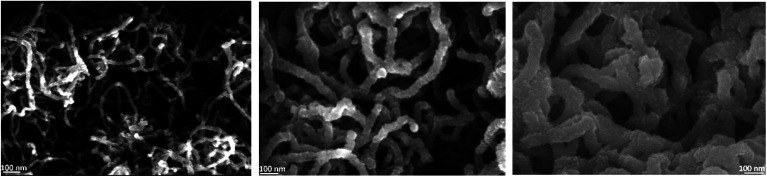
SEM of CNT–TiO_2_ structures with approximate diameters of 20 nm (left); 55 nm (middle) and 100 nm (right).

**Fig. 6:**
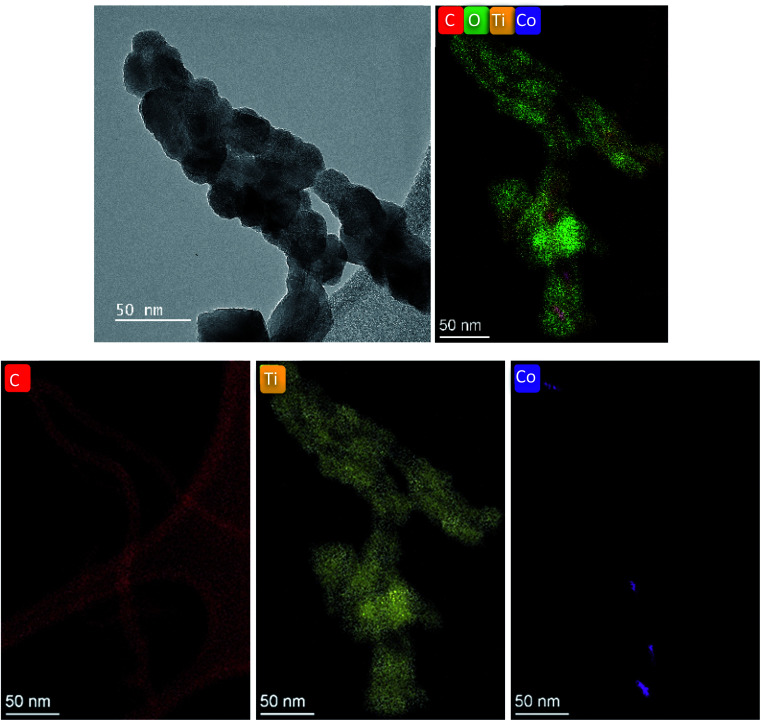
HRTEM of CNT–TiO_2_ sample confirming the core–shell structure formation.

TiO_2_ deposition at 175 °C was retained for films destined to the PEC measurements. The evolution of the morphology with the thickness of TiO_2_ is illustrated in Fig. 5. The preservation of the porous structure is noteworthy even after the deposition of a 45 nm thick TiO_2_ layer. High resolution TEM displayed in Fig. 6 shows the conformal coating of TiO_2_ layer on CNT confirming the formation of a core–shell structure.

The oxygen evolution reaction (OER) occurs at the anode involving holes, whereas the hydrogen evolution reaction (HER) occurs at the cathode involving electrons as shown in eqn (2). The OER requires an overpotential of 1.23 V_RHE_ that might be reduced when implementing photocatalysts such as TiO_2_.^14^

2 4OH^−^ → O_2_ + 2H_2_O + 4e^−^ (OER)

2H_2_O + 2e^−^ → H_2_ + 2OH^−^ (HER)2H_2_O → O_2_ + H_2_ (overall reaction at 1.23 V_RHE_)

Due to its n-type characteristics, TiO_2_ structures are used mostly as anode for the OER reaction. When n-type semiconductors, such as TiO_2_, are immersed in an electrolyte, an equilibrium is reached by the transfer of electrons from the semiconductor to the electrolyte. The formed space charge at the interface features an internal electric field and inhibits the further transfer of electrons to the electrolyte. However, upon light illumination, electron–hole pairs are generated, and the built-in electric field contributes to their separation. The photo-generated holes are drifted to the surface of the semiconductor and participate in the oxidation of adsorbed water molecules. Whereas, electrons are drifted to the bulk under the bias effect, and are further transported to the cathode.^6^ The O_2_ evolution reaction involves 4 holes along with the formation of O–O double bond. In principle, an overpotential beyond 1.23 V_RHE_ is required for the OER, while the overpotential required for H_2_ evolution is far smaller. Hence, OER is typically considered as a rate limiting step in the water splitting reaction.^41^ The extent of water oxidation is assessed by measuring the photocurrent density.

The investigated TiO_2_ was applied either on a planar Si substrate or on Si–CNTs. The surface area of CNT–TiO_2_ is significantly higher than the planar TiO_2_, which offers an extended interface with the electrolyte. The surface area in this case was approximated by combining the geometric thickness around the CNTs, as extracted from the SEM observation, and the weight gain as a result of TiO_2_ deposition (ESI†). Hereby the weight gain resulting from the ALD of TiO_2_ was assumed to be proportional to the real surface on which it is deposited. The surface area resulting from the 10 nm TiO_2_ deposition corresponds to 401 cm^2^ cm^−2^. The surface area varies from 401 to 209 cm^2^ cm^−2^ when the thickness of TiO_2_ is varied from 10 to 78 nm, which is related to the partial obstruction of the channels between CNTs. While the electrochemical reactivity of CNT–TiO_2_ might be related to the entire available TiO_2_–electrolyte interface area, the photoelectrochemical reactivity should take into consideration the light penetration depth and the competing light absorption by the CNTs. These effects reduce substantially the effective surface area of TiO_2_, which can be estimated using cyclic voltammetry with a varied scan rate in the negative potential range.^42^ Notwithstanding the relevance of the effective surface area, the photoelectrochemical characterization in this study refers to the geometric area, as the sun light is the factor that triggers the reactivity. The results displayed here correspond to illumination with a flux of 1 sun.

**Fig. 7:**
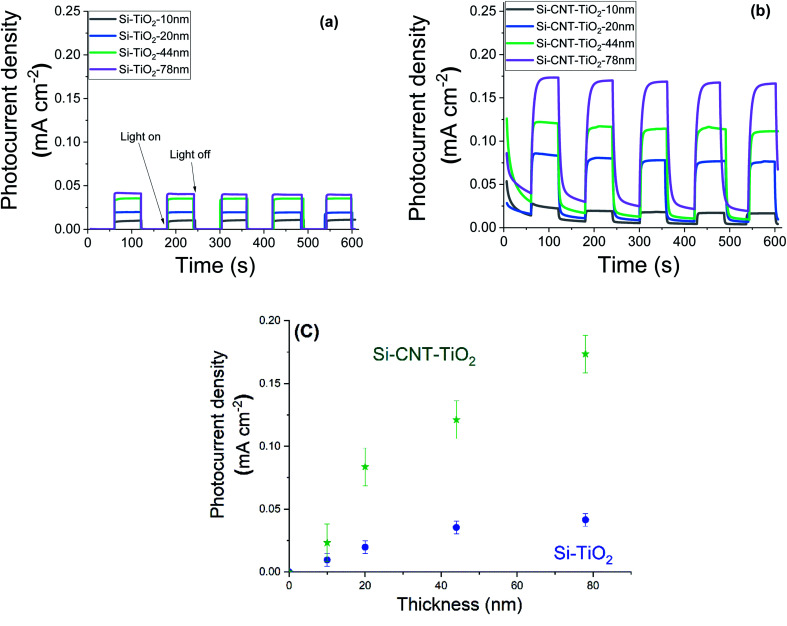
Photocurrent density of bare TiO_2_ (a) and CNT–TiO_2_ (b), under dark and light conditions with an illustration of the effect of TiO_2_ thickness (c).

Steady state chronoamperometry measurement was performed at a bias potential of 1.23 V_RHE_ to assess the photocurrent generated during intermittent illumination periods. The current is normalised to the geometric area and the results related to pristine CNTs and bare silicon substrates are depicted in the ESI (Fig. S1†). The results reveal a photocurrent in the order of 1–2 μA cm^−2^. Fig. 7 shows the equivalent results with the application of various thicknesses of TiO_2_. Upon illumination the current density raised quickly from 0 to reach an equilibrium plateau in the case of TiO_2_ films on silicon substrates. However, CNT–TiO_2_ core–shell structure features a residual dark current density that reduced gradually. Here the dark current is attributed to the presence of surface charge trapping, for which the suppression needs an extended time in the electrolyte. The current density response to light switching of thick TiO_2_ on CNT is slow relative to the grown TiO_2_ on silicon substrate, which is also associated with charge trapping that is emphasized by the large surface area.^43^ Trapped charges might either witness a transfer across the interface or a recombination.^44^

The CNT–TiO_2_ core–shell structure features a photocurrent density of 0.17 mA cm^−2^ at 1.23 V_RHE_, which is 425% higher than bare TiO_2_ with a similar thickness (*i.e.* 0.04 mA cm^−2^). One of the primary limitations of TiO_2_ material is the short diffusion length of minority charge carriers, ∼10 to 100 nm,^45^ which is associated with the high recombination rate. This hinders holes (h^+^) from reaching the interface with the electrolyte. In case of CNT–TiO_2_ core–shell structure, the photogenerated electrons are likely to witness a transfer to the CNTs, which would diminish the risk of bulk recombination in TiO_2_. In both cases the photocurrent density value increased with the thickness of TiO_2_. Photocurrent density is mostly affected by the photogenerated charge that depends on the thickness. The photocurrent density increases with the TiO_2_ thickness on Si up to 45 nm, where a plateau above is observed in contrast to the grown TiO_2_ on CNT.

**Fig. 8:**
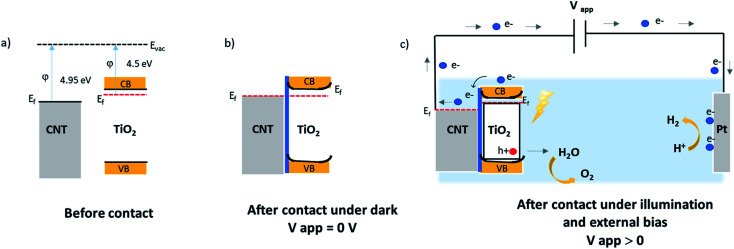
Illustrative band diagram of MWCNT–TiO_2_ before (a); after contact at equilibrium dark conditions (b); and under illumination with an external bias (c).

N-type silicon/n-type TiO_2_ heterostructure promotes the photogenerated charge carrier recombination at the interface.^46^ The electrically resistive undoped silicon substrates were used in this study, which forces a lateral electron transport in the TiO_2_ phase resulting in an enhanced recombination of the charge carriers. In the case of CNT–TiO_2_ structures, bulk recombination is likely to be limited due to the high electron conductivity of CNTs, and the short distance that electrons should cross in TiO_2_ prior being collected. Here the work function of 4.95 eV (ref. 47) and 4.5 eV (ref. 48) were reported for metallic CNTs and for TiO_2_ respectively. Therefore, the contact between CNT and TiO_2_ favours the transfer of electrons towards the CNT π-system.^49^ The transfer of electrons from TiO_2_ to CNT leads to the attainment of an equilibrium by balancing the Fermi levels. A built-in electric field at this interface inhibits the further flow of electrons towards CNT, forming a Schottky barrier with negatively charged metallic multiwalled CNTs. The height of this barrier can be reduced however by applying an external bias,^50^ enabling the flow of photogenerated electrons from TiO_2_ to CNT as illustrated in Fig. 8. The TiO_2_–electrolyte interface will also feature a built-in electric field that further enhances the photogenerated charge separation by driving the photogenerated holes towards the surface.

**Fig. 9:**
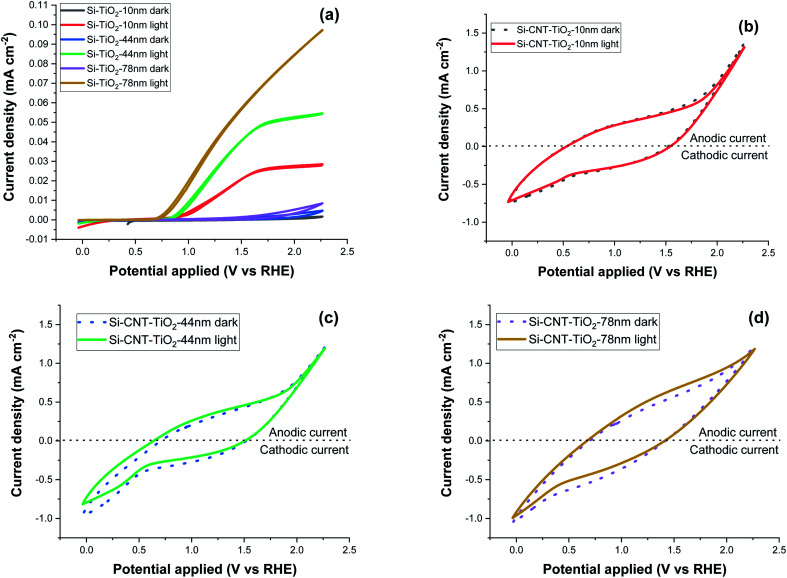
Cyclic voltammetry measurement at 0.1 V s^−1^ scan rate, of Si–TiO_2_ (a), Si–CNT–TiO_2_ samples (b–d).

Cyclic voltammetry (CV) measurement was performed in the 0–2.2 V potential range with a 0.1 V s^−1^ scan rate and the results are displayed in Fig. 9. Si–TiO_2_ samples, Fig. 9a, show negligible dark current densities throughout the potential range. Increasing the thickness of TiO_2_ induces a perceptible increase of the dark current indicating the occurrence of electrocatalytic reactions. Exposing the surface to solar radiation brings a relatively prominent increase of the current density. The last features a significant increase with the bias potential and with the thickness of TiO_2_. The onset potential is defined as the bias potential at which the anodic photocurrent starts to increase. This onset potential under light exposure is observed at 0.96 V for Si-10 nm TiO_2_ sample and it shifts further negatively to reach 0.82 V with a TiO_2_ thickness of 78 nm.

The CV measurements of CNT–TiO_2_ samples, Fig. 9b–d, show significant forward & reverse dark currents that increase with the bias potential, giving rise to a hysteresis behaviour. A marginal current density increase is observed upon illumination and the hysteresis characteristics are retained. This behaviour is qualitatively like the one observed for non-coated CNTs (figure S1b†). The current density obtained from the voltammetry measurement might be categorised into faradaic and non-faradaic current. The faradaic response is due to the redox-reaction with a transfer of electrons at the electrode–electrolyte interface and the capacitive current is related to the charging of the electrochemical double layer formed at the electrode–electrolyte interface.^51^ The electrochemical water oxidation occurs over pristine CNT sites at high overpotential.^52,53^ The steady state current density at 1.23 V_RHE_ was measured with a periodic exposure to light (Fig. S1a†). While the steady dark current is high for pristine CNT, the sensitivity to light exposure is relatively marginal. This variation in current density in steady state and at 0.1 V s^−1^ scan rate shows the presence of a large non-faradaic capacitive current with a marginal faradaic contribution. This behaviour was presumably attributed to the electrocatalytic reactivity over TiO_2_, partially covered CNTs or cobalt decorated CNTs. The potential contribution of electrocatalysis will be prominent as it would concern the total CNT–TiO_2_ layer, which contrasts with the photo-electrocatalytic reaction that is limited to the penetration depth of light. The carrier charge density of TiO_2_ was assessed as a function of the thickness using the Si–TiO_2_ model system and the Mott–Schottky analysis (Fig. S2 in the ESI†). A decrease from 3.8 × 10^16^ to 2.6 × 10^15^ cm^−3^ was noticed when increasing the thickness from 10 to 78 nm, which was related to a lower density of grain boundaries. With a known density of charge carrier, the impedance spectroscopy was implemented to assess the photo-electrochemically effective surface area as a function of the thickness of TiO_2_ on CNTs. The results reveal indeed that only 7% of the available surface is photo-electrochemically effective with the TiO_2_ thickness of 10 nm. This percentage rises to 36% with thicker TiO_2_ film (78 nm).

The investigation of the CNT–TiO_2_ system reveals advantages leading to an enhancement of the photocurrent with a factor exceeding 400%. This includes the enhancement of the surface area and the withdrawing of electrons from TiO_2_. The thorough analysis indicates however several limitations with further optimization potential. Among these aspects we might emphasize the band alignment between the electron collector and TiO_2_, competing for light absorption and the contribution of parasitic capacitive current. It is worth mentioning that the oxidative selective removal of CNTs leads to a semi-transparent film of randomly oriented TiO_2_ nanotubes with high interface area with the electrolyte. This structure does not suffer from the competing light absorption from the CNTs but is missing the fast electron conduction channel. The resulting weak photocurrent, not shown, evidences that the enhanced electrons transfer and their transport in the CNTs outweighs their competing light absorption. This result shows also that the increase of the surface area resulting from the use of CNT support is not the factor exclusively dominating the photoelectrochemical response of the core–shell structure.

## Conclusion

CNT–TiO_2_ nanocomposite coatings have been grown in this study, and their PEC characterization was performed. Single step thermal CVD process was used for the growth of CNT film, resulting in a randomly oriented CNTs, which were used for the ALD growth of the oxide layer (TiO_2_). Anatase phase has been grown *via* the hydrolysis of titanium tetra-isopropoxide that exhibits a constant growth rate 0.056 nm per cycle between 140 and 190 °C. The crystallinity of the film improves, however, with the temperature in this range.

CNT–TiO_2_ core–shell configuration outperforms bare TiO_2_ films in terms of PEC water splitting rate at a constant potential bias. The improvement in the photocurrent was attributed to the enhancement of the TiO_2_–electrolyte interface and the electrons-removal. Here CNTs act as nano-structuring support and as an electron transport channel.

## Funding

This research was funded by the Luxembourg National Research Fund through the MASSENA Pride program, the grant number: FNR PRIDE/MASSENA/15/10935404. A. Huerta acknowledges FNR for the Industrial Fellowship PLASPEROX 19/13711983.

## Conflicts of interest

The authors declare no conflict of interest.

## Supplementary Material

RA-011-D1RA05723E-s001
