# Supplementary material for: CNT–TiO2 core–shell structure: synthesis and photoelectrochemical characterization
Source: RSC Adv. 2021 Oct 8;11(52):33169–78. doi: 10.1039/d1ra05723e (PMC9042234; doi:10.1039/d1ra05723e)
Supplement: RA-011-D1RA05723E-s001 [file RA-011-D1RA05723E-s001.pdf]

Electronic Supplementary Material (ESI) for RSC Advances.
This journal is © The Royal Society of Chemistry 2021

CNT-TiO_2 core-shell structure: synthesis and photoelectrochemical characterization.

Vasu prasad PRASADAM, Ali Margot HUERTA FLORES, Naoufal BAHLAWANE

Material Research and Technology Department, Luxembourg Institute of Science and Technology, Rue du Brill, L-4422 Belvaux, Luxembourg

Chrono-amperometry measurement of pristine CNT and bare silicon was performed at 1.23 V_{RHE} under intermittent light illumination. Silicon shows, Figure S1a, a weak dark current (2 10^{-5} mA/cm^2) relative to CNTs (5 10^{-3} mA/cm^2). The dark current in the case of CNT-TiO_2 is attributed to the electrocatalytic activity of decorated CNTs with traces of cobalt, used as a catalyst for their growth. A photocurrent of 1 µA cm^{-2} was measured for Si, while pristine CNT features a photocurrent of 2 µA cm^{-2}. The dark current density was subtracted from the current density under illumination to assess the photocurrent. It is worth mentioning that both photocurrent contributions are marginal in respect to that of coated samples with TiO_2.

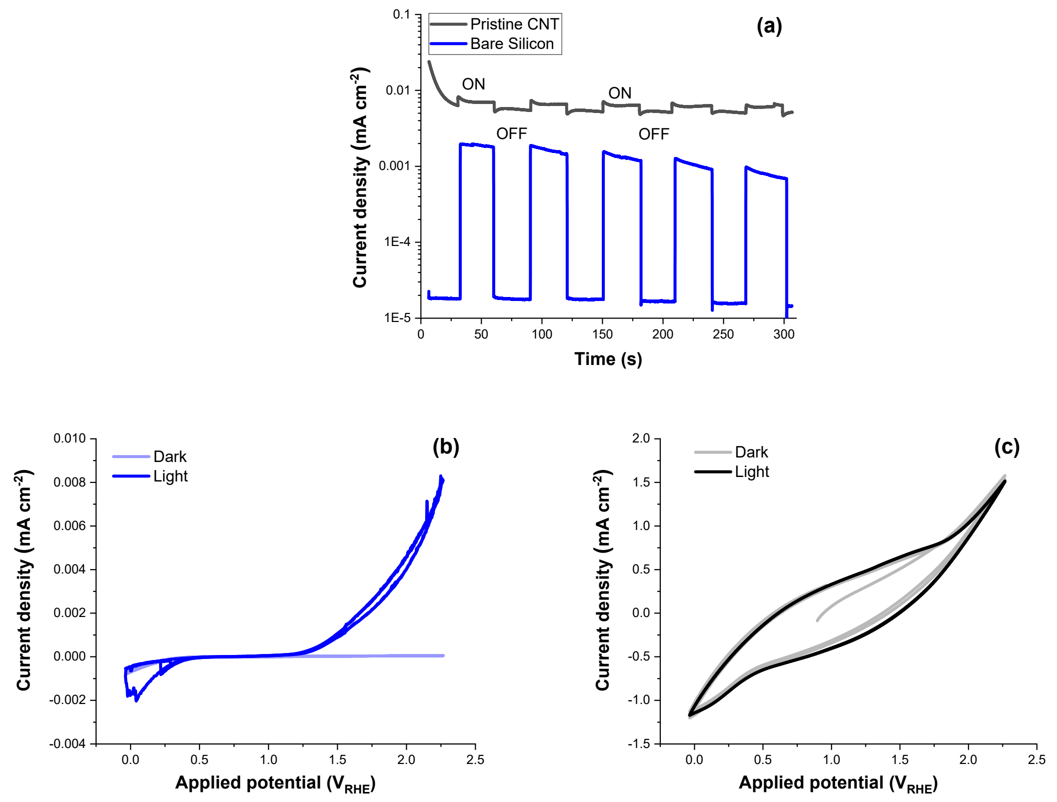

Figure S1: Chronoamperometric measurement at 1.23 V_{RHE} for pristine CNT and bare silicon (a); and the cyclic voltammetry at 0.1 V/s scan rate for bare silicon (b) and pristine CNT (c).

Cyclic voltammetry on silicon (Figure S1b) has shown no significant sensitivity to the applied potential in the dark, however a substantial increase is observed with the applied potential under illumination to reach 8 μA/cm^2 at 2.25 V_{RHE}. This magnitude is very small relative to pristine CNT that shows a broad hysteresis with a high dark-current and a relatively weak sensitivity to illumination (Figure S1c). The non-faradaic capacitive current likely dominates the dark response, which is in line with the implementation of CNT as an electrode material for supercapacitors.

Mott-Schottky analysis was performed based on the equation 1 for the Si-TiO_2 configuration.

$$\frac{1}{c^2} = \left(\frac{2}{\epsilon \cdot \varepsilon_0 . e \cdot N_d \cdot A^2}\right) \cdot \left(V - V_{fb} - \frac{K_B \cdot T}{e}\right)$$
Eq 1

where, ε is the dielectric constant (for anatase TiO_2, ε =48), ε_0 is the permittivity of vacuum, e the electron charge, N_d the charge carrier density, C is the space charge capacitance, V is the applied potential and V_{fb} is the flat band potential. The charge carrier density was extracted from the slope of the Mott-Schottky plot shown in figure S2 given by equation 2.

$$N_d = \left(\frac{2}{\epsilon \cdot \varepsilon_0 \cdot e \cdot A^2}\right) \cdot \left(\frac{dV}{d\left(\frac{1}{c^2}\right)}\right)$$
Eq 2

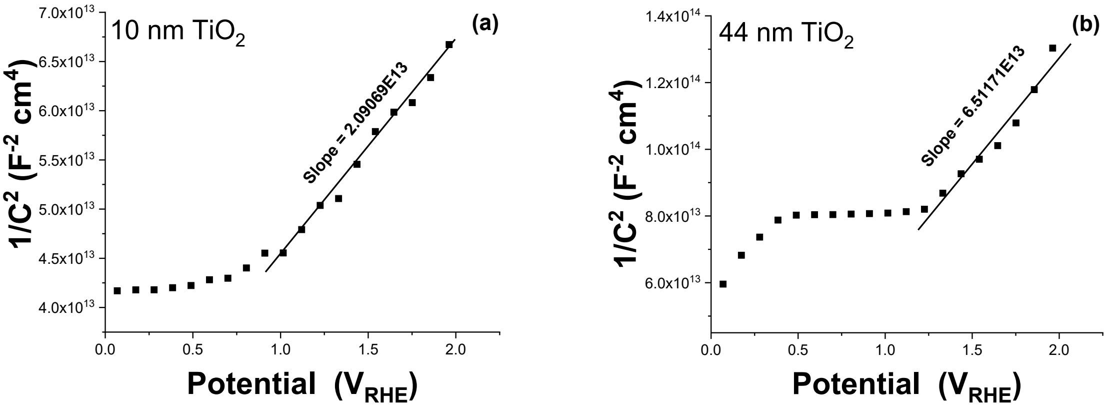

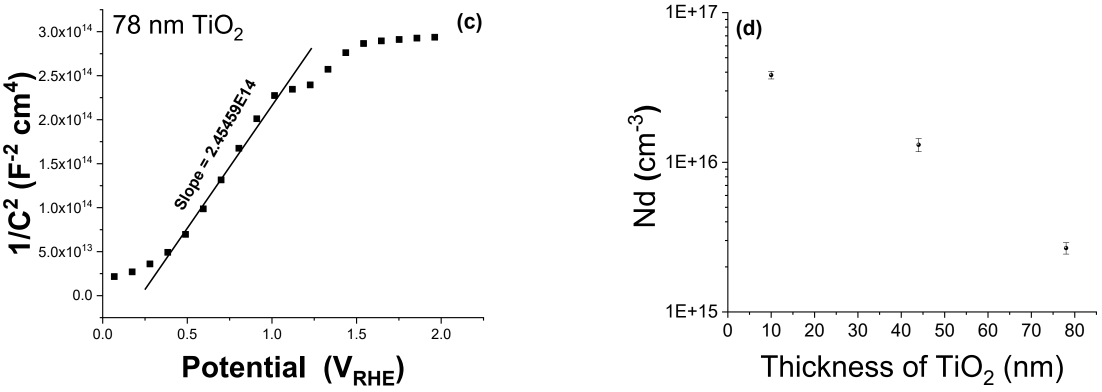

Figure S2: Mott-Schottky curves obtained at 10Hz for Si-TiO_2 (a-c) samples featuring different TiO_2 thicknesses. Donor density extracted from the Mott Schottky analysis are displayed in the panel (d).

The overall charge density decreases with the thickness, which might be attributed to a decrease in the concentration of the electroactive grain boundaries[1]. Thicker films allow for the growth of larger grains, which decreases the density of grain boundaries that act as donor-defective sites by the aggregation of oxygen vacancies (V_o) and titanium interstitials (Ti_i).

The open circuit potential (OCP) was measured using a three-electrode setup and the potential difference was assessed between the working electrode (Si-TiO_2 or Si-CNT-TiO_2) and the reference electrode (Ag/AgCl). The photo-potential is defined as the difference in steady state between the open circuit potential in dark and light i.e. $(OCP_{dark} - OCP_{light})$. Here the OCP_{light} shifts more to the negative potential, relative to OCP_{dark}, Figure S3, due to the generation and re-distribution of photo-charge carriers. The Si-TiO_2 samples show an increase of both OCP_{dark} and OCP_{light} with the increase of the TiO_2 thicknesses from 10 nm to 80 nm. The resulting photo-potential for TiO_2 shows relatively higher values for the 10 nm and 80 nm thick films. It is worth noting that the SEM inspection shows a partial coverage and the evolution of surface roughening for these films successively (Figure S4).

The OCP_{dark} in the case of CNT-TiO_2 samples is insensitive to the presence and to the thickness of TiO_2. While the Si-CNT shows a barely measurable sensitivity to illumination, Si-

$CNT-TiO_2$ samples feature a clear decrease of the OCP upon illumination, however the TiO_2 thickness does not significantly impact the response. In fact, the $Si-CNT-TiO_2$ samples feature two contributions due to the $CNT-TiO_2$ and TiO_2-electrolyte junctions. Their opposing effect might explain the low photo-potential relative to the $Si-TiO_2$ samples.

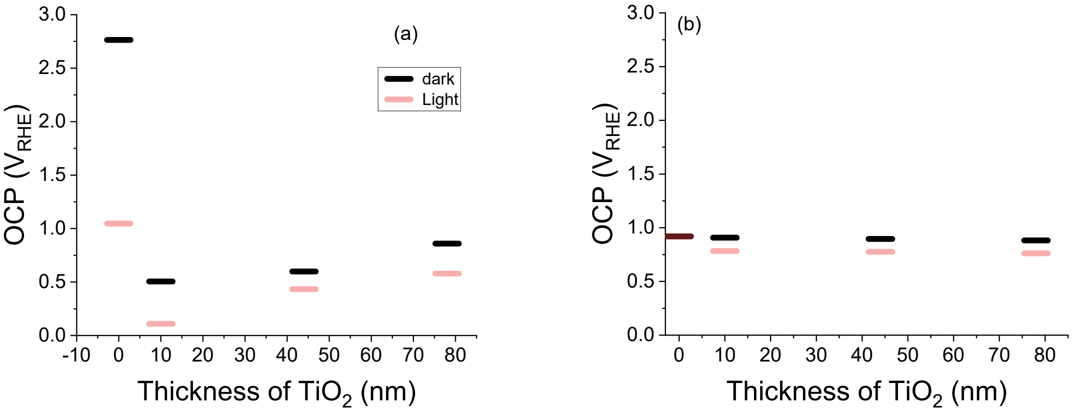

Figure S3: Open-circuit potential under dark & light for $Si-TiO_2$ (a), $Si-CNT-TiO_2$ (b) for various TiO_2 thicknesses.

The SEM inspection of planar TiO_2 on silicon substrates shows generally a smooth morphology composed of crystals that are far larger than the thickness of the film. The ultrathin film (10 nm) features a partial coverage, while the thickest (80 nm) features a surface roughening.

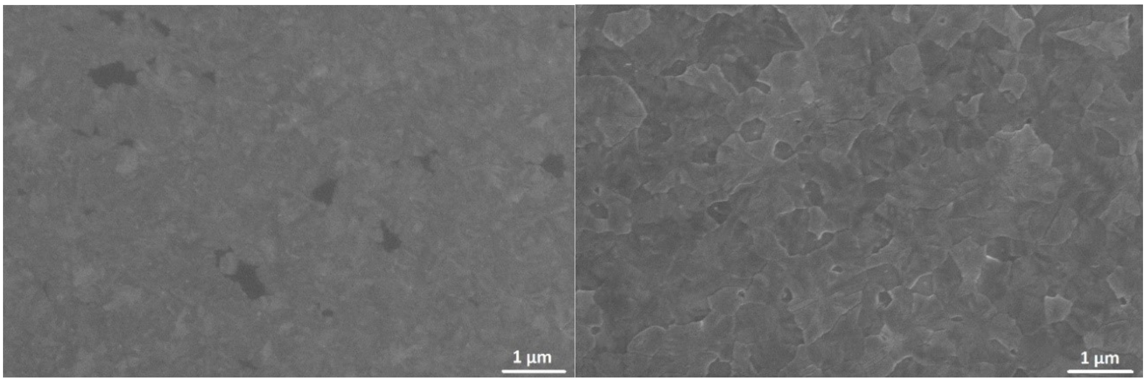

(a) (b)

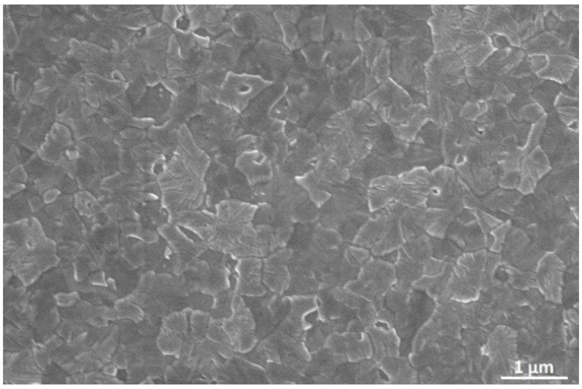

(c)

Figure S4: SEM images of Si-TiO_2 with TiO_2 thickness of 10 nm (a), 44 nm (b), 78 nm (c)

The mass of TiO_2 coating on CNTs was assessed by weighing the samples before and after the ALD process. Taking advantage of the highly conformal nature of the ALD made TiO_2 films, and assuming their full density, it is reasonable to consider that the weight gain is proportional to the available surface for the growth. The weight gain on planar silicon was calculated considering the density of the film and the thickness obtained by ellipsometry. The obtained data are depicted in figure S5.

The planar sample retains a surface area of nearly 1 cm^2 regardless of the TiO_2 thickness, whereas films grown on CNT feature a surface area evolving from 401 to 209 cm^2 as a function of the TiO_2 thickness from 10 to 78 nm. The pristine CNTs feature a diameter of 12 nm and the assessed surface corresponds to the total TiO_2 surface area.

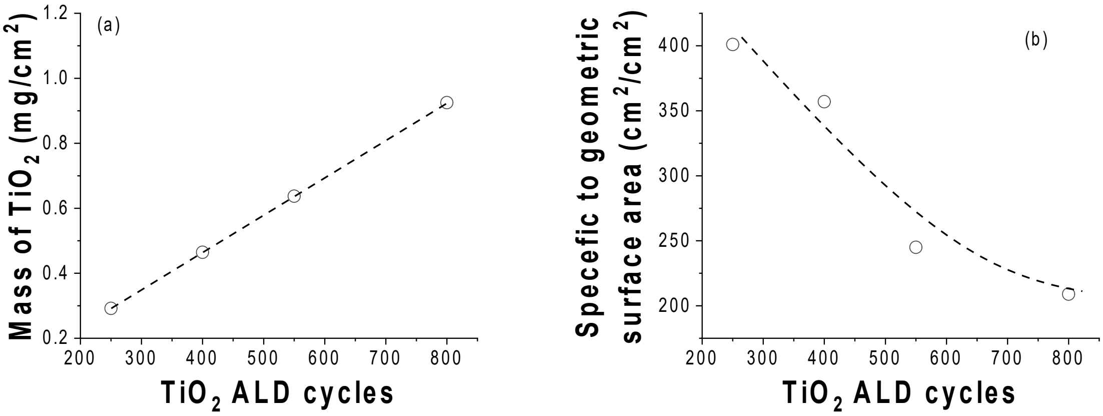

Figure S5: The mass of TiO_2 on CNT/Si (a) and the extracted surface area relative to the geometric surface (b).

The effective surface area is identified as the part participating to the photoelectrochemical reaction. The last was estimated using the impedance spectroscopy on CNT-TiO_2. From the equation 2, effective surface area (A) of CNT-TiO_2 is estimated by considering a similar carrier density value as for the grown TiO_2 with equivalent thickness on Si. Here we consider the

charge carrier density of Si-TiO_2 and CNT-TiO_2 to be similar because the growth method and kinetics of TiO_2 on Si and CNT are identical. In this case the calculation leads to 29 cm^2 as the effective surface area for TiO_2 with a thickness of 10 nm. The unfavourable real/effective surface area (0.07) hints at the combined effect of limited light penetration depth and the competing absorption on CNTs. The eventual presence of a double junction in the CNT-TiO_2 contributes presumably to this discrepancy. This competition is lower for thicker TiO_2 film leading to a ratio of 0.358 for a thickness of 78 nm.

Reference

1. Sellers MCK, Seebauer EG. Manipulation of polycrystalline TiO 2 carrier concentration via electrically active native defects. *J Vac Sci Technol A Vacuum, Surfaces, Film*. 2011;29(6):061503. doi:10.1116/1.3635373
